# Optimization of Cassava Starch/Onion Peel Powder-Based Bioplastics: Influence of Composition on Mechanical Properties and Biodegradability Using Central Composite Design

**DOI:** 10.3390/foods14142414

**Published:** 2025-07-08

**Authors:** Assala Torche, Chouana Toufik, Fairouz Djeghim, Ibtissem Sanah, Rabah Arhab, Maria D’Elia, Luca Rastrelli

**Affiliations:** 1 Laboratory of Protection of Ecosystems in Arid and Semi-Arid Zones (Eco-Sys), Department of Biological Sciences, Faculty of Natural and Life Sciences, Kasdi Merbah University, Ouargla 30000, Algeria; chouana.toufik@univ-ouargla.dz; 2 © Laboratoire de Nutrition et Technologie Alimentaire (L.N.T.A), Institut de la Nutrition, de l’Alimentation et des Technologies Agro-Alimentaires (INATAA), Université Frères Mentouri Constantine 1, Constantine 25017, Algeria; fairouze.djeghim@umc.edu.dz; 3 Laboratoire de Génie Biologique Valorisation et Innovation des Produits Agroalimentaires Institut ISTA-Ain M’Lila, Université Larbi Ben M’hidi Oum El-Bouaghi, Oum El-Bouaghi 04000, Algeria; sanah.ibtissem@univ-oeb.dz; 4 Laboratoire de Recherche en Sciences Alimentaires, Formulation, Innovation, Valorisation et Intelligence Art Ficielle (SAFIVIA), Institut de la Nutrition, de l’Alimentation et des Technologies Agro-Alimentaires (INATAA), Université Frères Mentouri Constantine 1, Constantine 25017, Algeria; 5 Department of Nature and Life Sciences, Larbi Ben M’Hidi University, Oum El Bouaghi 04000, Algeria; rarhab@clermont.inra.fr; 6 National Biodiversity Future Center (NBFC), 90133 Palermo, Italy; mdelia@unisa.it; 7 Department of Pharmacy, University of Salerno, Via Giovanni Paolo II, 132, Fisciano, 84084 Salerno, Italy; 8 Dipartimento di Scienze della Terra e del Mare, University of Palermo, 90133 Palermo, Italy

**Keywords:** biodegradable plastics, cassava starch, onion peel powder, central, composite design, soil burial test

## Abstract

Synthetic plastic pollution represents a major global concern, driving the search for sustainable and biodegradable packaging alternatives. However, many biodegradable plastics suffer from inadequate mechanical performance. This study aimed to develop a biodegradable film based on cassava starch, incorporating onion peel powder (OPP), a byproduct rich in quercetin derivatives, as a reinforcing agent and plasticized with crude glycerol. A Central Composite Design (CCD), implemented using Minitab 19, was employed to investigate the effects of starch (60–80%) and OPP (0–40%) content on the mechanical properties and biodegradability of the resulting bioplastics. Three optimized formulations were identified according to specific performance criteria. The first formulation, containing 72.07% starch and 21.06% OPP, was optimized for maximum tensile strength while maintaining target values for elongation and biodegradability. The second, composed of 77.28% starch and 37.69% OPP, was optimized to enhance tensile strength and biodegradability while minimizing elongation. The third formulation, with 84.56% starch and 27.74% OPP, aimed to achieve a balanced optimization of tensile strength, elongation, and biodegradability. After a 30-day soil burial test, these formulations exhibited weight loss percentages of 31.86%, 29.12%, and 29.02%, respectively, confirming their biodegradability. This study optimized the mechanical and biodegradability properties of cassava starch-based bioplastics using statistical modeling. The optimized formulations show potential for application in sustainable food packaging.

## 1. Introduction

The increasing environmental burden caused by petroleum-based plastics has driven global efforts to identify sustainable alternatives. Among these, biodegradable biopolymers derived from renewable resources have emerged as a promising solution, particularly in food packaging applications. In this context, starch-based films have received growing attention due to their low cost, film-forming ability, and biodegradability. However, their mechanical properties and water sensitivity often limit their practical applications, prompting the need for reinforcing strategies using natural fillers or bioactive byproducts [1]. Such materials could help reduce waste accumulation, limit reliance on fossil fuels, and lower carbon dioxide emissions. Biodegradable plastics, derived from renewable plant-based resources such as starch and cellulose, can be naturally decomposed by microorganisms due to their biological origin. Chitosan, starch, and cellulose are among the most extensively studied biopolymers for food packaging applications [2]. Starch, in particular, is favored due to its availability, low cost, and biodegradability. However, starch-based plastics often exhibit brittleness and inferior mechanical properties compared to synthetic polymers, which limits their applicability in packaging [3].

The onion (*Allium cepa*) is a widely cultivated and consumed vegetable across the globe. According to FAO data, the global annual harvest amounts to 93.23 million tons, with China being the leading producer. Algeria ranks as the world’s tenth-largest producer, contributing 1.76 million tons. Despite their culinary value, onions generate a considerable amount of waste, particularly peels, which are typically discarded due to their strong odor, rendering them unsuitable for animal feed or fertilizer. Conventional disposal methods such as landfilling or incineration are both costly and environmentally damaging. This is especially wasteful considering the high content of valuable fibers and phytochemicals present in onion peels. In a recent study, we characterized the flavonoid profiles and biological activities of onion skins from two traditional varieties cultivated in southern Italy. The phytochemical analysis revealed that these by-products are particularly rich in flavonols and anthocyanins, such as quercetin, its glycosides, and cyanidin 3-glucoside, and exhibit significant antioxidant and anti-diabetic activities, as well as proliferative effects on human fibroblasts. These findings clearly demonstrate the potential of onion skin as a source of bioactive compounds for applications in industrial, nutraceutical, and cosmetic sectors [4]. Onion peel waste, therefore, represents an underutilized resource that can be transformed into value-added products. Given the environmental burden posed by conventional plastics, the transformation of food waste into biodegradable packaging offers a promising and sustainable alternative. Utilizing onion peel powder in biodegradable film formulations may contribute to addressing both ecological and economic issues related to food waste and plastic pollution.

The rationale for combining cassava starch and onion peel powder lies whichn their complementary properties. Cassava starch is widely used in biodegradable films due to its availability and film-forming capability, but it tends to exhibit low tensile strength and poor flexibility. Onion peel powder, on the other hand, is rich in dietary fiber and flavonoids such as quercetin, which may act as reinforcing agents and influence biodegradation through antimicrobial and structural effects. The central hypothesis of this study is that the incorporation of onion peel powder into a cassava starch matrix will improve the mechanical strength of the resulting film and modulate its biodegradability. This effect is expected to result from the interaction between the bioactive-rich, fibrous OPP and the polysaccharide-based starch matrix. To improve the performance of biodegradable plastics, Response Surface Methodology (RSM) is commonly applied as an optimization tool [5]. This statistical approach employs mathematical modeling to identify optimal conditions by analyzing the interactions among multiple input variables. The Central Composite Design (CCD) is a widely used RSM technique, particularly effective for evaluating the influence of formulation variables on material properties. In this study, biodegradable composites were developed using cassava starch and onion peel powder. A Central Composite Design, whichmplemented via Minitab 19, was used to optimize the composition with respect to three key performance indicators: tensile strength, elongation, and biodegradability. To the best of our knowledge, few studies have explored the fabrication of bioplastics using onion peel powder in a starch-based matrix, particularly in combination with a Central Composite Design (CCD) for formulation optimization.

## 2. Materials and Methods

### 2.1. Standards and Materials

Ultrapure water (18 MΩ) was obtained using a Milli-Q purification system (Millipore, Bedford, MA, USA). MS-grade water (H_2_O), methanol (MeOH), and acetonitrile (CH_3_CN) were supplied by Romil (Cambridge, UK). Analytical grade MeOH, CH_3_CN, formic acid (HCOOH), and absolute ethanol (EtOH) were supplied by Carlo Erba reagents (Milan, Italy). MS-grade formic acid (HCOOH), quercetin (purity ≥ 95%), isorhamnetin (purity ≥ 95% HPLC), kaempferol (purity ≥ 97%), and protocatechuic acid (purity 95%) were also provided.

### 2.2. OPP Preparation

Onion peels were collected from a local market in the Oum El Bouaghi district. In the laboratory, any adhering dirt or debris was removed by thoroughly washing the peels with water. To facilitate drying, the peels were cut into smaller pieces and left to dry under ambient conditions until a constant weight was achieved. Once dried, the peels were ground using a ball mill (Fritsch Pulverisette 7 premium line), and the resulting powder was sieved through a 45 µm mesh to obtain uniformly sized particles. OPP was stored in unsealed glass bottles at room temperature and pressure until further use.

### 2.3. OPP Extraction

Exhaustive extraction was performed using ultrasound-assisted solid–liquid extraction (UAE) at 25 °C in a thermostat-controlled ultrasound bath (Labsonic LBS2, Treviglio, Italy) at the frequency of 20.0 kHz. Dried *Allium cepa* skins were extracted using aqueous EtOH (70% *v*/*v*) and a matrix/solvent ratio of 1:20 (3 × 30 min). At each extraction cycle, after centrifugation 10 min at 9000× *g*, the extracts of each variety were pooled, filtered (Whatman No. 1 filter), and freeze-dried (freeze dryer Alpha 1–2 LD, Christ, Berlin, Germany), after the removal of the organic solvent under vacuum at 40 °C in a rotary evaporator (Rotavapor R-200, Buchi Italia s.r.l, Cornaredo, Italy). Extraction yields of 10.4% were obtained. The extract was then used for further chemical characterization.

### 2.4. Quantitative Analysis by UHPLC-UV

Quantitative analyses were performed using a Dionex Ultimate 3000 UHPLC system (Thermo Fisher Scientific, San Jose, CA, USA), equipped with an Ultimate 3000 RS pump, autosampler, column compartment, and variable wavelength detector. Chromatographic separation was carried out on a Kinetex C18 UHPLC column (2.1 × 50 mm, 1.7 µm; Phenomenex, Torrance, CA, USA). The mobile phases consisted of MS-grade water (A) and acetonitrile (B), both containing 0.1% formic acid (HCOOH). The gradient elution program was as follows: 0–3 min, isocratic at 2% B; 3–5 min, linear gradient from 2% to 13% B; 5–9 min, isocratic at 13% B; 9–12 min, linear gradient from 13% to 18% B, held for 1 min; 13–17 min, linear gradient from 18% to 30% B; 17–21 min, linear gradient from 30% to 50% B; 21–22 min, linear gradient from 50% to 98% B, followed by washing and column re-equilibration for 5 min. The flow rate was set at 600 μL min^−1^, the column temperature was maintained at 30 °C, and the injection volume was 5 μL. UV chromatograms were monitored at 295 and 365 nm, based on the absorption maxima of the phenolic compounds. Quantification was performed using an external standard calibration method. Calibration curves were generated using mixtures of two commercially available reference standards, protocatechuic acid and quercetin, across six concentration levels (6.25–100 μM), with triplicate injections for each level. The calibration curves showed good linearity (R^2^ > 0.998) over the tested range. The identification of compounds was supported by the strong agreement of their retention times and UV-Vis spectral features with those of commercial or isolated standards, as well as with previously characterized compounds.

Since not all phenolic compounds identified in the extract were available as commercial standards, quantification of quercetin derivatives was performed using the calibration curve of quercetin. Results were expressed as quercetin equivalents (mg/100 g dry matter). When necessary, extracts were diluted to ensure analyte concentrations fell within the dynamic range of the calibration curves.

### 2.5. Starch–OPP Film Preparation

**Figure 1:**
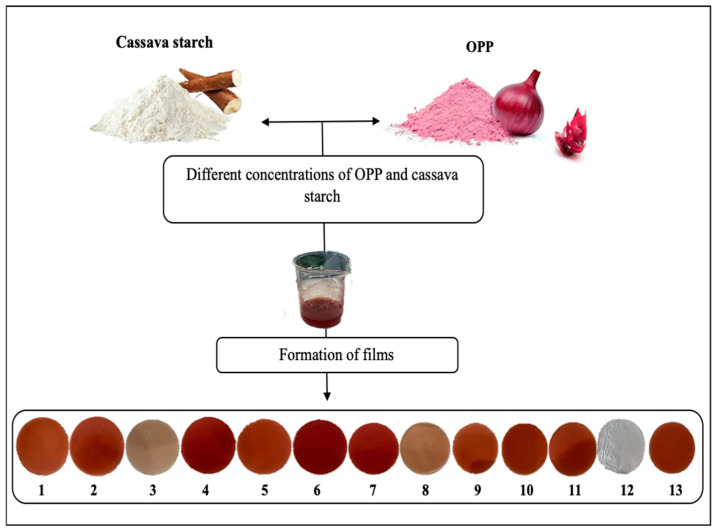
Scheme explaining the fabrication process of biodegradable films made of starch and OPP.

Starch–OPP films were produced using the casting method, following the procedure described by Oliveira Filho et al. [6], with slight modifications. Preliminary tests confirmed that starch-based films plasticized with glycerol (2.5% *v*/*v*) and cast via solvent evaporation formed continuous and flexible films, consistent with literature reports. However, these films displayed limited mechanical strength and were highly sensitive to moisture. Therefore, onion peel powder (OPP) was incorporated as a natural reinforcing filler to improve mechanical integrity and modulate biodegradation performance. Film-forming solutions (FFSs) were prepared based on the experimental design described in Table 1, incorporating cassava starch and OPP at ratios determined through preliminary optimization studies. The percentages of cassava starch and onion peel powder (OPP) listed in Table 1 represent their relative proportions within the dry biopolymer matrix. These values do not account for the total weight of the film-forming solution, which also includes distilled water and glycerol (2.5% *v*/*v*) as a plasticizer. To prepare the FFS, cassava starch was dissolved in an aqueous glycerol solution (2.5% *v*/*v*) and heated to 85 °C under continuous stirring at 280 rpm for 30 min. Simultaneously, OPP was dispersed in distilled water at 25 °C and stirred for 10 min. The OPP dispersion was then combined with the starch solution and stirred for an additional 2 min to ensure homogeneity. A volume of 25 mL of the resulting mixture was poured into Petri dishes (ϕ = 10 cm) and dried in an incubator at 25 °C for 24 h (Figure 1). The dried films were then peeled and stored in a desiccator under ambient conditions until further analysis.

### 2.6. Experimental Design

A Central Composite Design (CCD) with two factors, cassava starch (*X*_1_) and onion peel powder (*X*_2_), was employed to optimize the formulation of the biodegradable plastic. This design included five levels for each factor (−α, −1, 0, +1, +α), allowing for a comprehensive evaluation of both individual and interactive effects on the selected response variables.

The content of cassava starch (*X*_1_) ranged from 60% to 100%, while onion peel powder (*X*_2_) varied from 0% to 40%. These ranges were established based on preliminary experimental screening.

The design comprised 13 experimental runs (Table 1). The following response variables were selected as critical indicators of suitability for packaging applications: tensile strength (*Y*_1_), elongation at break (*Y*_2_), and biodegradability (*Y*_3_).

The relationship between the independent variables and each response was modeled using the second-order polynomial Equation (1):

*Y* = b_0_ + b_1_*X*_1_ + b_2_*X*_2_ + b_11_*X*_1_^2^+ b_22_*X*_2_^2^ + b_12_*X*_1_*X*_2_ (1)

In this model, b_0_ represents the predicted response at the center point (0,0); b_1_ and b_2_ denote the linear effects of cassava starch and onion peel powder, respectively; b_11_ and b_22_ represent the quadratic effects; and b_12_ accounts for the interaction between the two factors [7].

### 2.7. Mechanical Test

The mechanical properties of the films, specifically tensile strength (TS) and elongation at break (EB), were evaluated using a texture analyzer (TA.Zwick/Roell), following a modified procedure based on Santos et al. [8]. Rectangular film strips measuring 50 mm × 20 mm were prepared for testing. The tests were conducted at an initial crosshead speed of 10 mm/min, with a gauge length (distance between grips) set at 50 mm. The instrument software (testXpert V12.0) continuously recorded the force–displacement data throughout the test. Tensile strength was determined as the maximum force sustained before breakage, while elongation at break was calculated as the percentage of strain at the point of rupture.

### 2.8. Biodegradability Test

The biodegradability of the films was evaluated based on weight loss, following a modified version of the method described by Afshar et al. [9]. Square film samples (2 cm × 2 cm) were buried at a depth of 5 cm in soil-filled pots and watered daily to maintain moisture levels. The soil used was collected from an agricultural field and consisted of rhizosphere soil, which is naturally rich in microorganisms. Soil moisture was adjusted and maintained at 70% of field capacity (70% FC), and the burial tests were carried out at a controlled temperature of 27 °C. Although microbial profiling was not performed, rhizosphere soils typically harbor diverse microbial populations averaging around 10^8^ CFU/g. All samples were buried in the same batch of soil to ensure uniform microbial exposure. After 30 days of incubation under ambient conditions, the samples were carefully removed, gently cleaned to eliminate any adhering soil, and dried before weighing. The percentage of weight loss was calculated using Equation (2), where Wi is the initial dry mass and Wf is the final dry mass after burial.

(2) $$Weight\;loss\left(\%\right)=\frac{Wi-Wf}{Wi}\times 100\%$$

Although visual disintegration was not always complete after 30 days, the test duration was standardized for all samples, and weight loss was used as a reliable quantitative metric for biodegradation, as per the adapted methodology.

### 2.9. Statistical Analysis

All statistical analyses, including response surface modeling and optimization, were performed using Minitab 19 software (Minitab Inc., State College, PA, USA). All experiments were conducted in triplicate, and the results are presented as mean ± standard deviation (SD). One-way analysis of variance (ANOVA) was employed to evaluate the effects of cassava starch and onion peel powder content on the mechanical properties and biodegradability of the bioplastic films. A significance level of *p* < 0.05 was used to determine statistical significance.

## 3. Results

### 3.1. Identification and Quantification of Phenolic Compounds by UHPLC-UV

Fourteen phenolic compounds were identified and quantified in the red onion peel extract using UHPLC-UV analysis. Compound identification was carried out by comparing UV absorption spectra, retention times, and chromatographic profiles with those previously reported (Table 2) [4]. Quantification was performed using an external standard calibration approach, employing commercially available standards including quercetin, kaempferol, isorhamnetin, and protocatechuic acid. Due to the unavailability of certain standards, quercetin derivatives were quantified using the quercetin calibration curve, and the results are expressed as quercetin equivalents (mg per 100 g dry matter). Despite the absence of mass spectrometric data in this study, the detailed analytical framework established in our previous work enabled the reliable identification and quantification of major phenolic constituents in the extract.

### 3.2. Effect of Cassava Starch and Onion Peel Powder on Tensile Strength of Film

The effect of cassava starch (*X*_1_) and onion peel powder (*X*_2_) on the tensile strength of the biodegradable films is summarized in Table 3. The tensile strength of the bioplastics varied significantly across the experimental runs, ranging from 0.52 Mpa to 3.87 Mpa. These values are consistent with the findings of Kechichian et al. [10], who reported similar tensile properties in cassava starch films containing natural antimicrobial agents, and those of dos Santos Caetano et al. [11], who observed comparable results with starch-based films enriched with natural compounds. The highest tensile strength was observed in the formulation containing 40% OPP, the highest concentration tested. This aligns with previous research highlighting the reinforcing role of onion peel derivatives in biopolymer matrices, including cellulose-based films [12] and protein-rich systems [13].

**Figure 2:**
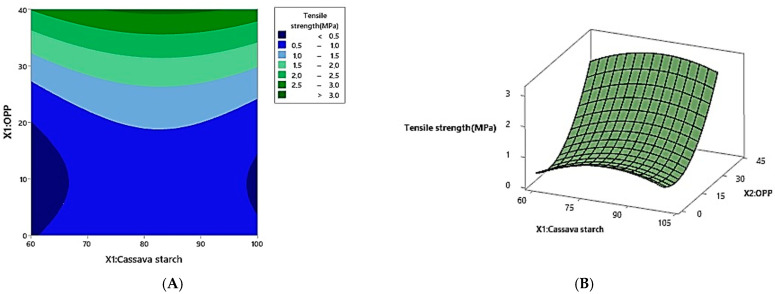
The effect of cassava starch (*X*1) and OPP (*X*2) on the tensile strength response; surface contour (**A**), and three-dimensional plots (**B**).

As expected, the starch–glycerol matrix yielded flexible films due to the plasticizing effect of glycerol. However, such films generally exhibit poor mechanical strength and low resistance to environmental stressors such as humidity and microbial exposure. The addition of onion peel powder (OPP) provided structural reinforcement, as evidenced by increased tensile strength across several formulations. This enhancement is attributed to the fibrous nature of OPP and its polyphenolic constituents, which likely promote matrix cohesion through hydrogen bonding and other intermolecular interactions. The synergistic effect of glycerol-induced flexibility and OPP-induced reinforcement constitutes a key innovation of the present study. As predicted by the regression model (Table 4), tensile strength was significantly (*p* < 0.05) influenced by both the linear and quadratic terms of onion peel powder (OPP) content. The coefficient of the first-order term in Equation (3) (after excluding non-significant terms) indicated that tensile strength increased with higher OPP content. The model explained 81.53% of the variability in the tensile strength response (R^2^ = 0.8153). The response surface plot (Figure 2) further corroborates these findings, demonstrating a positive correlation between OPP content and tensile strength. At lower OPP concentrations, tensile strength decreased due to factors such as poor compatibility between starch and OPP, disruption of the starch matrix, and increased brittleness. However, as the OPP content increased, tensile strength improved due to enhanced interfacial adhesion through hydrogen bonding and electrostatic interactions between hydroxyl groups of starch and phenolic compounds (e.g., quercetin) in onion peel powder. These interactions promote stronger cohesion within the matrix, contributing to reinforcement and improved resistance to deformation. This suggests the existence of an optimal OPP concentration at which tensile strength is maximized (Figure 2). The observed enhancement in tensile strength can be attributed to two primary factors: (1) the formation of strong intermolecular hydrogen bonds, which promote the development of a rigid and continuous network within the film matrix, and (2) the presence of numerous oxygen (O) and hydroxyl (OH) groups in the quercetin structure of OPP. These functional groups are capable of forming electrostatic interactions with active groups in both starch and OPP, thereby enhancing film cohesion and ultimately increasing tensile strength [14]. Similar reinforcement effects have also been observed with the incorporation of nano-starch and tannic acid-coated nano-starch, which enhanced mechanical strength by improving hydrogen bonding and interfacial compatibility within starch-based film matrices [15]. While the model effectively captures the overall trends in tensile strength, some deviations (e.g., between Runs 4 and 6) may also result from microstructural factors beyond composition. Establishing a definitive link between film composition and mechanical performance is challenging without detailed morphological or structural analysis. Variations in filler dispersion, porosity, and matrix crystallinity may significantly influence mechanical performance. This interpretation remains hypothetical, as it is inferred from compositional data and literature. Future studies should include structural techniques (e.g., FTIR spectroscopy, SEM imaging, or molecular modeling) to investigate the underlying structure–property relationships more directly.

### 3.3. Effect of Cassava Starch and Onion Peel Powder on Elongation of Film

The elongation of the bioplastics varied significantly across runs, ranging from 14.09% to 97.06% (Table 3). Similar trends were identified by Alqahtani et al. [16] and Ayyubi et al. [17] for films made from corn starch-based materials reinforced with date palm pits and chitosan/cassava starch/PVA/crude glycerol, respectively. The greatest elongation was observed in the run with the highest cassava starch content (100%), suggesting that cassava starch contributes to the bioplastic’s ductility.

**Figure 3:**
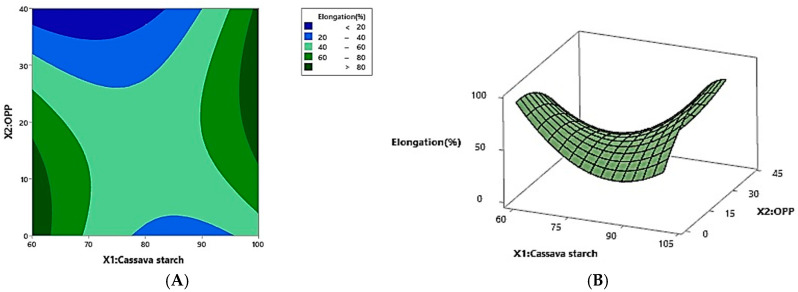
The effect of cassava starch (*X*1) and OPP (*X*2) on the elongation response: surface contour (**A**), and three-dimensional plots (**B**).

The regression model (Table 4) revealed that elongation was significantly influenced (*p* < 0.05) by both the linear and quadratic terms of cassava starch (*X*_1_) and onion peel powder (OPP) concentration (*X*_2_), as well as their interaction. The model explained 97.23% of the variation in elongation (R^2^ = 0.9723). The coefficients of the first-order term in Equation (4) (Table 5) indicate that elongation increased with cassava starch content but decreased with OPP content. Additionally, cassava starch exhibited a negative quadratic effect, while OPP showed a positive quadratic effect on film elongation (*p* < 0.05). A positive interaction between cassava starch and OPP also influenced film elongation (Table 3). The relationship between cassava starch, OPP content, and elongation is visualized in Figure 3. The elongation behavior observed across the experimental runs does not follow a linear trend with cassava starch content alone. For instance, although Run 10 exhibited the highest elongation (97.06%) with 100% starch, other runs with the same OPP level but lower starch concentrations (e.g., Run 9, 11, and 13 with 80% starch) showed considerably lower elongation values. Conversely, Run 2, with 60% starch and 20% OPP, exhibited a relatively high elongation of 72.07%. These findings suggest that elongation is governed by a complex interplay between starch and OPP concentrations, their physical compatibility, and possibly microstructural features. The statistical model (Equation (4)) accounts for these nonlinear interactions through interaction and quadratic terms.

Our films exhibited a reduction in elongation as the OPP content increased. This trend is consistent with findings by Adilah et al. [18] and Piñeros et al. [19], who reported reduced elongation with increased concentrations of mango kernel extract in films composed of soy protein isolate and fish gelatin, and rosemary in cassava starch films, respectively. Onion peel is a rich source of mono- and polysaccharides, including fructo-oligosaccharides, gelling pectin, and a low quantity of lignin-based dietary fiber, along with fragrant compounds exhibiting biological activity [20]. These carbohydrates likely act as plasticizers, contributing to the observed decrease in elongation [21]. The results of this investigation suggest that carbohydrates and bioactive compounds in OPP serve a plasticizing role in cassava starch films.

### 3.4. Effect of Cassava Starch and Onion Peel Powder on the Biodegradability of Film

**Figure 4:**
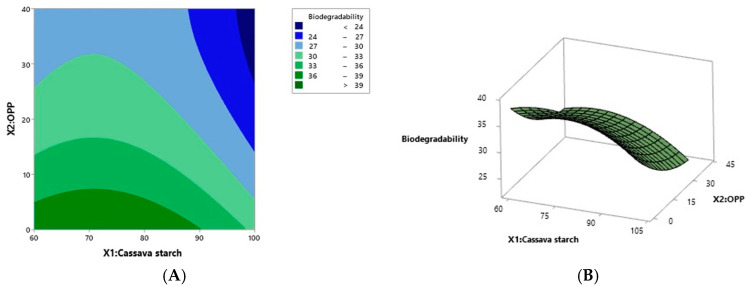
The effect of cassava starch (*X*1) and OPP (*X*2) on the biodegradability response: surface contour (**A**) and three-dimensional plots (**B**).

**Figure 5:**
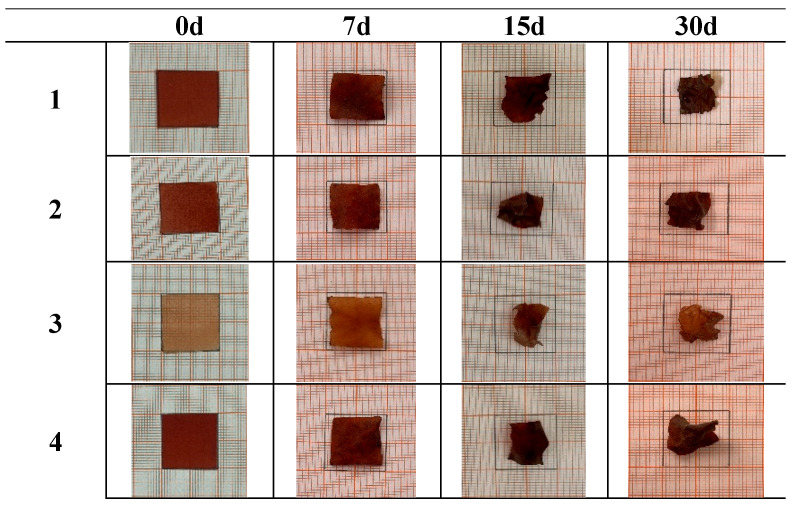

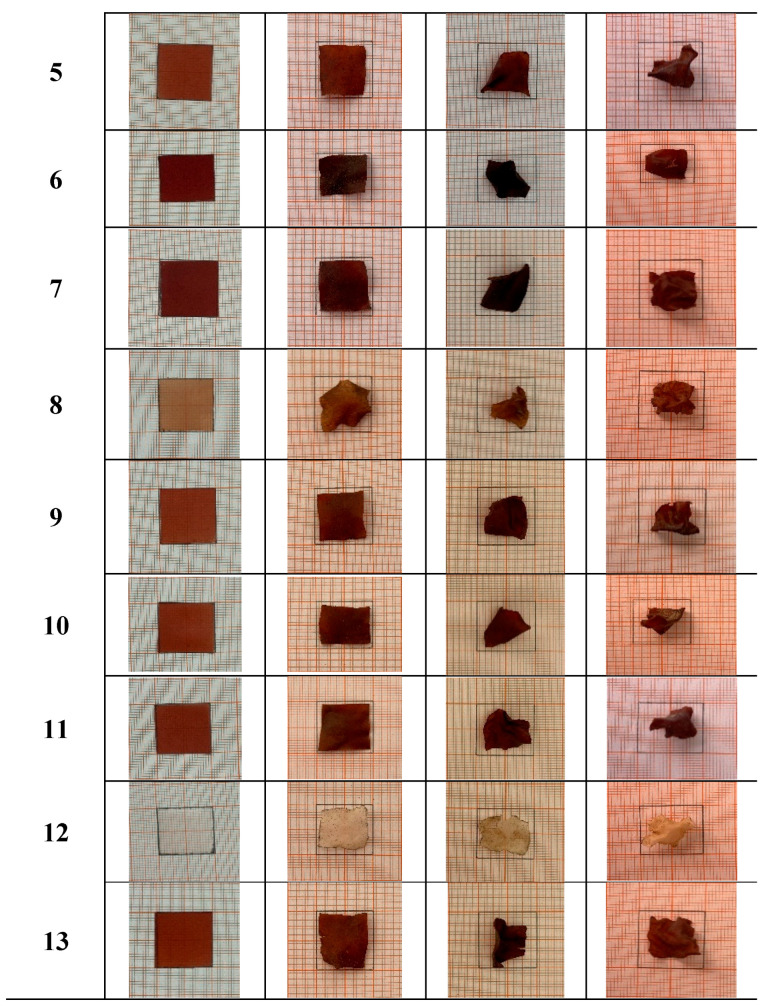
The appearance of 0, 7, 15, and 30-day degradation of the composite films.

The biodegradability of the bioplastics varied from 25.21% to 38.38% (Table 3, Figure 4). Interestingly, the highest biodegradability was observed in the run with the lowest onion peel powder content (0%), suggesting a potential negative impact of OPP on biodegradability. These results are consistent with earlier studies by Ayyubi et al. [17] and Romero et al. [22], which reinforce the significant impact of composition on the mechanical characteristics and biodegradability of eco-friendly films. The regression model (Table 4) indicated that biodegradability was significantly affected (*p* < 0.05) by both linear and quadratic terms of cassava starch (*X*_1_) and OPP content (*X*_2_). The model explained 95.42% of the variation in biodegradability (R^2^ = 0.9542). The coefficients from the first-order term in Equation (5) (Table 5) show that biodegradability decreased with increasing levels of both cassava starch and OPP. Additionally, cassava starch exhibited a negative quadratic effect, while OPP showed a positive quadratic effect on film biodegradability (*p* < 0.05). Figure 5 provides a visual representation of how cassava starch and OPP content relate to the biodegradability of the composite films. The images, spanning a 30-day degradation period, show that while initially the films are intact with diverse appearances, they generally undergo degradation characterized by shrinking size, shape distortion and fragmentation, increased brittleness and porosity, changes in surface texture, and sometimes color alterations. Importantly, the degree and speed of this breakdown differ among the various film formulations, indicating that the specific ratio of cassava starch to OPP significantly affects their biodegradability. The incorporation of onion peel powder (OPP) into the bioplastic matrix appears to moderately reduce the biodegradation rate. This outcome can be attributed to multiple factors. First, the phenolic compounds present in OPP, known for their antimicrobial activity, may inhibit the enzymatic function of soil microorganisms responsible for biodegradation. Second, the high cellulose content in OPP contributes to its structural resilience, making it less susceptible to microbial attack compared to starch. While starch is composed of glucose units linked by α-1,4 glycosidic bonds, which are readily hydrolyzed by microbial enzymes, cellulose consists of β-1,4 linkages, forming a more crystalline and recalcitrant structure that slows microbial degradation [13,23].

### 3.5. Optimization and Model Validation

The previous section developed mathematical models correlating starch and OPP content with the selected response variables. Building upon these models, this section focuses on optimizing the biodegradable composite composition. According to the literature [24], biodegradable films exhibiting tensile strength (TS) ranging from 1 to 10 MPa and elongation at break (EB) between 10% and 100% are considered satisfactory. Optimization was conducted based on specific criteria for each formulation. For Formula (1), the goal was to maximize tensile strength while maintaining target values for elongation and biodegradability. Formula (2) prioritized maximum tensile strength and biodegradability while minimizing elongation. Lastly, Formula (3) aimed to maximize all three properties: tensile strength, elongation, and biodegradability. These optimization goals were aligned with the requirements of food packaging applications, which demand high mechanical strength. Subsequent verification experiments, whose results are presented in Table 6, demonstrated close agreement between predicted and experimental values. Among all the tested formulations, Run 1 (80% cassava starch, 20% OPP) emerged as the most balanced in terms of mechanical integrity and biodegradation performance. With a tensile strength of 1.07 MPa, elongation at break of 48.7%, and biodegradability of 30.83%, this formulation offers a viable compromise between functional strength and environmental sustainability, making it a promising candidate for bio-based packaging applications.

## 4. Discussion

The results of this study demonstrate the potential of cassava starch-based biodegradable films incorporating onion peel powder (OPP) to enhance mechanical strength. However, the impact on biodegradability was variable and, in many cases, negative, especially at higher OPP concentrations, likely due to the antimicrobial and structural characteristics of OPP.

The formulations optimized via CCD show promising properties for sustainable food packaging applications. The compositional analysis of OPP by UHPLC/MS and HPLC/UV revealed the presence of phenolic compounds, particularly flavonoids such as quercetin derivatives, which are known to possess antioxidant and antimicrobial activity. These components may contribute to the observed increase in tensile strength and reduction in biodegradability, likely by reinforcing the matrix and inhibiting microbial enzymatic action during degradation. Several studies have highlighted the advantages of incorporating plant-based fillers, such as onion peel, into biodegradable films. For instance, Bayram et al. [25], explored the use of fruit and vegetable waste as fillers in biodegradable films and found that the incorporation of bioactive compounds from agricultural waste enhanced both the mechanical strength and antioxidant properties of the films. Similarly, our previous research has demonstrated the effectiveness of incorporating natural antioxidants and essential oils into biopolymeric matrices for active packaging applications, improving not only shelf life and microbial resistance but also the biodegradability of the materials [26,27,28].

This is consistent with our findings, where the incorporation of onion peel powder (OPP) into the cassava starch matrix improved tensile strength while maintaining a functional balance with elongation and biodegradability. The observed improvement in mechanical performance can be attributed to the rich phytochemical composition of OPP, particularly flavonoids such as quercetin [29], which, according to our UHPLC-MS analysis, are present in significant quantities. These polyphenolic compounds are known to act as reinforcing agents in biopolymer matrices, enhancing hydrogen bonding and intermolecular interactions. In addition to their mechanical role, polyphenols from onion peel also confer antioxidant and antimicrobial properties to the films. While antioxidants may support the stability of biopolymers by interacting with environmental stressors such as light and moisture, their antimicrobial activity may inadvertently inhibit the microbial colonization required for biodegradation. This dual effect likely explains the reduced biodegradability observed in our study, where the best-performing formulations exhibited weight loss percentages between 29% and 32% over a 30-day soil burial test.

Interestingly, while other studies [30] have reported that the addition of natural fillers accelerates biodegradation through enhanced microbial activity, our findings suggest that the phenolic structure of OPP may exert an inhibitory effect on microbial enzymes responsible for starch decomposition. This highlights the complex, concentration-dependent behavior of OPP in biodegradable film systems and underscores the importance of balancing mechanical enhancement with environmental performance in sustainable packaging materials.

In addition, the optimization process used in this study, based on CCD, allowed for the identification of formulations that strike an ideal balance between mechanical strength and biodegradability. The outcomes of this study are consistent with the findings of Rocha Gomes et al. [31] and Tafa et al. [32], who also utilized CCD to optimize the properties of bioplastics. The use of statistical design in bioplastic research is becoming increasingly important for the development of high-performance, sustainable materials. In comparison to conventional polypropylene-based films, which exhibit tensile strength values in the range of 25–40 MPa [33], the bioplastics developed in this study demonstrate lower mechanical performance. However, the maximum tensile strength achieved (3.87 MPa) and elongation (97.06%) are in line with starch-based biodegradable films reported in the literature and support their potential use in low-load packaging applications, such as fresh produce wrapping or single-use trays However, while the formulations developed in this study exhibit promising mechanical and biodegradation properties, further research is required to evaluate their performance under real-world conditions, particularly for food contact applications. Future investigations should focus on assessing the barrier properties of these films, including their water vapor permeability and moisture sensitivity, in order to comprehensively determine their suitability and long-term stability as sustainable food packaging materials.

## 5. Conclusions

Biodegradable plastics based on cassava starch and onion peel powder (OPP) were successfully developed using the casting method. A Central Composite Design was implemented and optimized through Minitab 19 software to achieve desirable mechanical properties, tensile strength, elongation at break, and acceptable biodegradability. The model’s predictions were validated experimentally, showing close agreement with observed values and confirming its reliability in guiding formulation optimization.

The incorporation of OPP improved the tensile strength of the films, while its effect on elongation and biodegradability appeared to be predominantly inhibitory, even at relatively low concentrations. This may be attributed to the presence of antimicrobial and structurally rigid compounds such as flavonoids and fiber, which interfere with microbial degradation processes.

These findings support the initial hypothesis that OPP modulates both the mechanical and degradability properties of starch-based films through complex, concentration-dependent mechanisms.

Overall, cassava starch/OPP-based bioplastics show promising potential as sustainable alternatives to petroleum-derived packaging materials. However, the study is limited to specific starch/OPP ratios. Future work should explore the inclusion of other natural additives or biopolymers to further enhance film functionality and environmental performance.

## Figures and Tables

**Table 1:** Central Composite Design matrix with coded and uncoded experimental values for cassava starch and onion peel powder content. Percentages refer to the dry biopolymer matrix and do not represent total formulation mass, which includes fixed amounts of water and glycerol.

Run	Coded Value *X*_1_ (Cassava Starch)	Coded Value *X*_2_ (OPP)	Uncoded *X*_1_ (%)	Uncoded *X*_2_ (%)
1	0.000	0.000	80.000	20.000
2	−1.414	0.000	60.000	20.000
3	1.000	−1.000	94.142	5.858
4	1.000	1.000	94.142	34.142
5	0.000	0.000	80.000	20.000
6	0.000	1.414	80.000	40.000
7	−1.000	1.000	65.858	34.142
8	−1.000	−1.000	65.858	5.858
9	0.000	0.000	80.000	20.000
10	1.414	0.000	100.000	20.000
11	0.000	0.000	80.000	20.000
12	0.000	−1.414	80.000	0.000
13	0.000	0.000	80.000	20.000

**Table 2:** Quantitative determination of phenolic compounds in red onion peel extract by UHPLC-UV.

N°	Compound Name	R(t) (min)	RDS (%)	mg/100 g DM ± RDS
1	Protocatechuic acid	1.2	1.8	135.8 ± 6
2	2-(3,4-Dihydroxybenzoyl)-2,4,6-trihydroxy-3(2H)-benzofuranone	6.0	3.9	116.5 ± 4
3	Quercetin-7,4′-diglycoside	6.5	7.0	63.5 ± 8
4	Quercetin-3,4′-diglycoside	6.9	5.5	74.3 ± 12
5	Quercetin-4′-glycoside	10.3	6.4	351.3 ± 8
6	Isorhamnetin-4′-glycoside	12.5	6.6	nq
7	Quercetin	13.5	7.6	510.4 ± 12
8	Kaempferol	16.4	2.0	nq
9	Isorhamnetin	16.7	2.6	nq
10	Quercetin dimer 4′-glycoside	17.1	1.9	44.1 ± 4
11	Quercetin dimer 4′-glycoside	18.2	0.8	43.3 ± 6
12	Quercetin dimer hexoside	18.5	2.5	nq
13	Quercetin dimer	19.4	0.8	110.3 ± 2
14	Quercetin trimer	20.7	2.4	62.8 ± 6

Note: nq = not quantified; R(t) = retention time; RDS% = relative standard deviation; DM = dry matter.

**Table 3:** Mechanical properties and biodegradability of biodegradable plastics formulated with varying starch and OPP concentrations.

Run	Cassava Starch (%)	Onion Peel Powder (%)	Tensile Strength (Mpa)	Elongation (%)	Biodegradability (%)
1	80.000	20.000	1.07 ± 0.017 ^e f^	47.63 ± 0.010 ^g^	30.83 ± 0.051 ^e f^
2	60.000	20.000	0.75 ± 0.005 ^i^	60.37 ± 0.015 ^d^	30.04 ± 0.040 ^f^
3	94.142	5.858	0.58 ± 0.010 ^j^	47.24 ± 0.045 ^h^	32.08 ± 0.060 ^c^
4	94.142	34.142	1.52 ± 0.011 ^b^	14.09 ± 0.030 ^l^	26.00 ± 0.50 ^h^
5	80.000	20.000	0.97 ± 0.015 ^g^	24.11 ± 0.051 ^k^	31.43 ± 0.030 ^c d e^
6	80.000	40.000	3.87 ± 0.011 ^a^	73.46 ± 0.060 ^b^	27.43 ± 0.015 ^g^
7	65.858	34.142	1.41 ± 0.020 ^c^	47.68 ± 0.026 ^g^	31.00 ± 1.15 ^d e f^
8	65.858	5.858	0.52 ± 0.015 ^k^	97.06 ± 0.065 ^a^	36.96 ± 0.015 ^b^
9	80.000	20.000	1.08 ± 0.010 ^d e^	52.85 ± 0.025 ^e^	30.69 ± 0.035 ^e f^
10	100.000	20.000	1.11 ± 0.015 ^d^	38.15 ± 0.035 ^j^	25.21 ± 0.020 ^h^
11	80.000	20.000	1.11 ± 0.015 ^d^	43.73 ± 0.015 ^i^	32.04 ± 0.037 ^c d^
12	80.000	0.000	0.88 ± 0.015 ^h^	47.63 ± 0.010 ^g^	38.38 ± 0.170 ^a^
13	80.000	20.000	1.03 ± 0.005 ^f^	60.37 ± 0.015 ^d^	32.20 ± 0.10 ^c^

*p*-value: *p* < 0.0001. Data are mean values ± standard deviations. Values in each column with different letters (a, b, c, d, e, f, g, h, i, j, k, l) are significantly different (*p* < 0.05). The CCD matrix included repeated runs with identical formulations to allow for statistical replication, which enabled the estimation of pure error and enhanced the accuracy of the regression model.

**Table 4:** Regression-coded coefficients for the second-order polynomial equation representing the relationship between the responses and process variables.

Response	Equation		R^2^
*Y*_1_: Tensile Strength (Mpa)	*Y*_1_ = −6.56 + 0.184 *X*_1_ – 0.051 *X*_2_ + 0.002494 *X*_2_^2^	Equation (3)	0.8153
*Y*_2_: Elongation (%)	*Y*_2_ = 681.3 – 14.97 *X*_1_ – 4.44 *X*_2_ + 0.08638 *X*_1_^2^ – 0.05974 *X*_2_^2^ + 0.0776 *X*_1_*X*_2_	Equation (4)	0.9723
*Y*_3_: Biodegradability (%)	*Y*_3_ = −1.1 + 1.132 *X*_1_ – 0.440 *X*_2_ – 0.00798 *X*_1_^2^ + 0.00522 *X*_2_^2^	Equation (5)	0.9542

**Table 5:** Coefficients and *p*-values for the relationship between the composition of starch and OPP concentration with tensile strength, elongation, and biodegradability of the film.

Variables	Tensile Strength (Mpa)	Elongation (%)	Biodegradability (%)
Coefficient	1.055	48.04	31.439
*p*-value	0.002	0.000	0.000
*X*_1_ (Cassava Starch)	0.085	5.72	−2.089
*p*-value	0.629	0.010	0.001
*X*_2_ (Onion Peel Powder)	0.757	−8.83	−3.440
*p*-value	0.003	0.001	0.000
*X*_1_^2^ (Cassava Starch^2^)	−0.224	17.28	−1.596
*p*-value	0.254	0.000	0.005
*X*_2_^2^ (Onion Peel Powder^2^)	0.499	−11.95	1.045
*p*-value	0.028	0.000	0.033
*X*_1_*X*_2_ (Interaction)	0.012	15.52	−0.030
*p*-value	0.959	0.000	0.956

**Table 6:** Prediction values and research results for three formulas of biodegradable film.

Formula		Cassava Starch (%)	Onion Peel Powder (%)	Tensile Strength (MPa)	Elongation (%)	Biodegradability (%)
1	Prediction	72.066	21.0563	0.99579	48.8895	31.8590
	Verification	72.066	21.0563	0.9754 ± 0.020	48.732 ± 0.253	31.835 ± 0.076
2	Prediction	77.2803	37.6878	2.7547	14.1122	29.1211
	Verification	77.2803	37.6878	2.713 ± 0.015	14.103 ± 0.015	29.114 ± 0.022
3	Prediction	84.5616	27.7373	1.62493	46.0143	29.0246
	Verification	84.5616	27.7373	1.619 ± 0.070	46.001 ± 0.002	29.018 ± 0.009

## Data Availability

The original contributions presented in the study are included in the article, further inquiries can be directed to the corresponding author.
